# *Glossogyne tenuifolia* Extract Increases Nitric Oxide Production in Human Umbilical Vein Endothelial Cells

**DOI:** 10.3390/ph14060577

**Published:** 2021-06-17

**Authors:** Chin-Feng Hsuan, Thung-Lip Lee, Wei-Kung Tseng, Chau-Chung Wu, Chi-Chang Chang, Tsui-Ling Ko, Ya-Ling Chen, Jer-Yiing Houng

**Affiliations:** 1 Department of Internal Medicine, Division of Cardiology, E-Da Hospital, Kaohsiung 82445, Taiwan; calvin.hsuan@msa.hinet.net (C.-F.H.); lip1969@hotmail.com (T.-L.L.); arthurtseng@seed.net.tw (W.-K.T.); 2 Department of Internal Medicine, Division of Cardiology, E-Da Dachang Hospital, Kaohsiung 82445, Taiwan; 3 School of Medicine, College of Medicine, I-Shou University, Kaohsiung 82445, Taiwan; 4 School of Medicine for International Students, College of Medicine, I-Shou University, Kaohsiung 82445, Taiwan; ed101779@edah.org.tw (C.-C.C.); kate819b@isu.edu.tw (T.-L.K.); 5 Department of Internal Medicine, Division of Cardiology, National Taiwan University Hospital, Taipei 100225, Taiwan; chauchungwu@ntu.edu.tw; 6 Department of Obstetrics & Gynecology, E-Da Hospital/E-Da Dachang Hospital, Kaohsiung 82445, Taiwan; igiolal2011@gmail.com; 7 Department of Nutrition, I-Shou University, Kaohsiung 82445, Taiwan; 8 Department of Chemical Engineering, I-Shou University, Kaohsiung 84001, Taiwan

**Keywords:** *Glossogyne tenuifolia*, nitric oxide, endothelial NO synthase, human umbilical vein endothelial cell, atherosclerosis

## Abstract

The vascular nitric oxide (NO) system has a protective effect in atherosclerosis. NO is generated from the conversion of L-arginine to L-citrulline by the enzymatic action of endothelial NO synthase (eNOS). Compounds with the effect of enhancing eNOS expression are considered to be candidates for the prevention of atherosclerosis. In this study, extracts from the aerial, root, and whole plant of *Glossogyne tenuifolia* (GT) were obtained with ethanol, *n*-hexane, ethyl acetate (EA), and methanol extraction, respectively. The effects of these GT extracts on the synthesis of NO and the expression of eNOS in human umbilical vein endothelial cells (HUVECs) were investigated. NO production was determined as nitrite by colorimetry, following the Griess reaction. The treatment of HUVECs with EA extract from the root of GT and *n*-hexane, methanol, and ethanol extract from the aerial, root, and whole plant of GT increased NO production in a dose-dependent manner. When at a dose of 160 μg/mL, NO production increased from 0.9 to 18.4-fold. Among these extracts, the methanol extract from the root of GT (R/M GTE) exhibited the most potent effect on NO production (increased by 18.4-fold). Furthermore, using Western blot and RT–PCR analysis, treatment of HUVECs with the R/M GTE increased both eNOS protein and mRNA expression. In addition, Western blot analysis revealed that the R/M GTE increased eNOS phosphorylation at serine^1177^ as early as 15 min after treatment. The chemical composition for the main ingredients was also performed by HPLC analysis. In conclusion, the present study demonstrated that GT extracts increased NO production in HUVECs and that the R/M GTE increased NO production via increasing eNOS expression and activation by phosphorylation of eNOS at serine^1177^.

## 1. Introduction

Atherosclerotic cardiovascular disease is the leading cause of morbidity and mortality worldwide [1]. Atherosclerosis is a chronic process involving complex mechanisms. Endothelium function is a regulator of vascular homeostasis. It responds to shear stress of blood flow to induce endothelial-dependent vasodilatation [2,3]. The major vasodilative substance is nitric oxide (NO), which is produced and released from the endothelium [2,4]. NO is generated in endothelial cells from its precursor L-arginine by the enzymatic action of endothelial NO synthase (eNOS). Endothelium-derived NO diffuses into the underlying smooth muscle cells and induces vasodilatation by activating quanylate cyclase [2,5]. Besides the vasodilatation effect, endothelial NO exerts anti-thrombotic and anti-atherosclerotic properties by inhibiting platelet adhesion and aggregation, leukocyte adhesion and infiltration to the vascular wall, and the proliferation of vascular smooth muscle cells. The influx and oxidation of low-density lipoprotein are also inhibited by NO [6].

Endothelial dysfunction is the earliest sign of atherosclerosis and is present in the preclinical stage [2]. The impairment of endothelium-dependent vasodilatation is present before vascular structural change [4]. The endothelial dysfunction of microcirculation also contributes to myocardial ischemia [7]. Studies have shown that endothelial function may predict the risk of cardiac events and may be a useful prognostic indicator of cardiovascular disease [8,9]. The major feature of endothelial dysfunction is impaired NO bioavailability, which can be the result of either increased degradation by superoxide under oxidative stress or reduced NO production by eNOS [10]. In atherosclerotic vessels, the expression of NADPH oxidase is increased and superoxide production is augmented [11,12]. Superoxide may react with NO to form peroxynitrite, reducing its bioactitivity. eNOS signaling requires several steps for NO production, including the interruption of the inhibitory interaction of caveolin-1 with eNOS, binding of the activator calmodulin to eNOS, phosphorylation of eNOS at Ser^1177^ to increase its activity, binding of the substrate L-arginine to eNOS, and binding of the cofactor 5,6,7,8-tetrahydrobipterin (BH_4_) [2]. Interruption in any step of eNOS signaling will reduce NO production and result in endothelial dysfunction.

Vascular NO has a protective role in atherosclerosis. Materials designed to improve the NO bioavailability may be a potential remedy to prevent atherosclerosis. Some natural substances, such as vitamins and polyphenols, have been shown to increase the expression of eNOS and inhibit the action of NADPH oxidase, thereby increasing the production of NO [13,14,15,16]. All of these chemicals are good antioxidants.

*Glossogyne tenuifolia* (GT, Hsiang Ju grass), a perennial plant distributed in Southern Asia and Australia, is also native to Penghu, Taiwan. It has long been used for making a traditional antipyretic, hepatoprotective, and anti-inflammatory herbal tea on Penghu Island [17]. Indeed, GT has been demonstrated to have anti-inflammatory [18,19,20] and strong antioxidant activity [21,22,23]. It was also proven to have antiviral [19] and anti-osteoclastogenic [24] properties in addition to its cytotoxicity on several human cancer cell lines [21]. Our previous study demonstrated that GT can protect against endothelial injury by suppressing the formation of free reactive oxygen species [25], and exert anti-atherosclerotic effect by inhibiting monocytes’ adhesion to endothelium [26]. Moreover, GT attenuates the proliferation and migration of vascular smooth muscle cells, which are essential in the pathogenesis of various vascular diseases such as atherosclerosis and restenosis [27]. Due to these effects, GT may have the potential to increase the production of NO, and it is therefore important to investigate the effect of GT extracts on endothelial cells.

The present study was designed to examine the effects of ethyl acetate (EA), *n*-hexane, ethanol, and methanol extracts of the aerial, root, and whole plant of GT on the regulation of NO production in human umbilical vein endothelial cells (HUVECs) and to evaluate their potential as a preventive of endothelial dysfunction.

## 2. Results

### 2.1. Cell Viability

At first, the cytotoxicity of each extract of GT on HUVECs was assessed using the 3-(4,5-dimethylthiazol-2-yl)-2,5-diphenyltetrazolium bromide (MTT) assay. The cell viability at 24 h did not decrease after incubation with each extract of GT at concentrations up to 160 μg/mL (Supplementary Figure S1), indicating that each extract of GT was not cytotoxic to HUVECs within the concentration ranges tested.

### 2.2. Effects of Extracts of GT on NO Production in HUVECs

**Figure 1:**
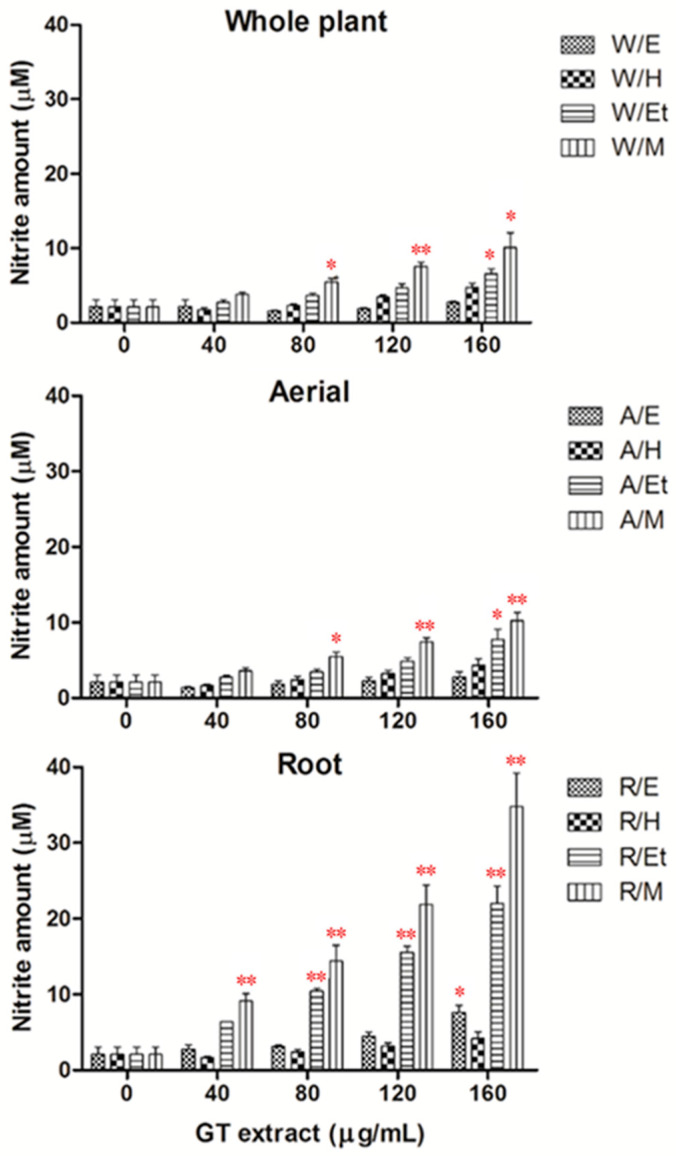
Effects of different extracts from the whole plant, aerial and root of GT on NO production in HUVECs. HUVECs were treated with each extract for 24 h. NO production was determined as the formation of nitrite. Data were obtained from five replicate experiments, and are expressed as mean ± standard deviation. W, whole plant; A, aerial; R, root; E, EA extract; H, hexane extract; Et, ethanol extract; M, methanol extract. A significant difference from the vehicle was indicated as * *p* < 0.05, ** *p* < 0.01, (Student’s *t*-test).

**Figure 2:**
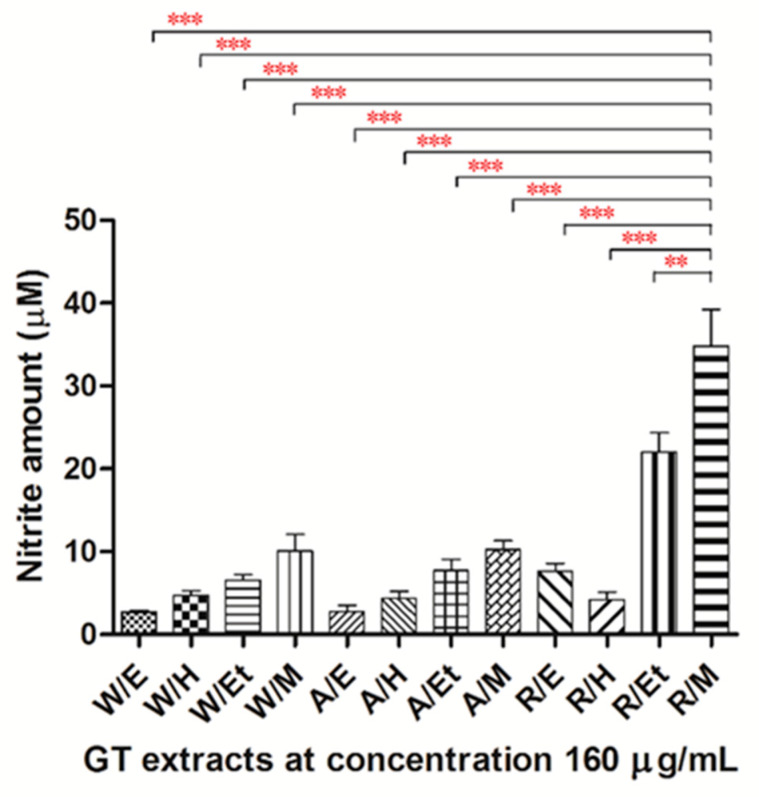
One-way ANOVA analysis and post-hoc analysis for NO production on various extracts. HUVECs were treated with each extract for 24 h. NO production was determined as the formation of nitrite. Data were obtained from five replicate experiments, and are expressed as mean ± standard deviation. W, whole plant; A, aerial; R, root; E, EA extract; H, hexane extract; Et, ethanol extract; M, methanol extract. A significant difference was indicated as ** *p* < 0.01 or *** *p* < 0.001.

In the present study, nitrite was assayed as a measure of NO synthesis. Treatment of HUVECs with the EA extract from the root of GT, methanol, and ethanol extract from the aerial, root, and whole plant of GT for 24 h significantly increased NO production in a dose-dependent manner (Figure 1). When at the dose of 160 μg/mL, the NO production increased from 0.9 to 18.4-fold more than the vehicle group. Among these extracts, the methanol extract from the root of GT (R/M GTE) exhibited the most potent effect on NO production in HUVECs (Figure 2).

### 2.3. Effect of the R/M GTE on eNOS Protein Expression

**Figure 3:**
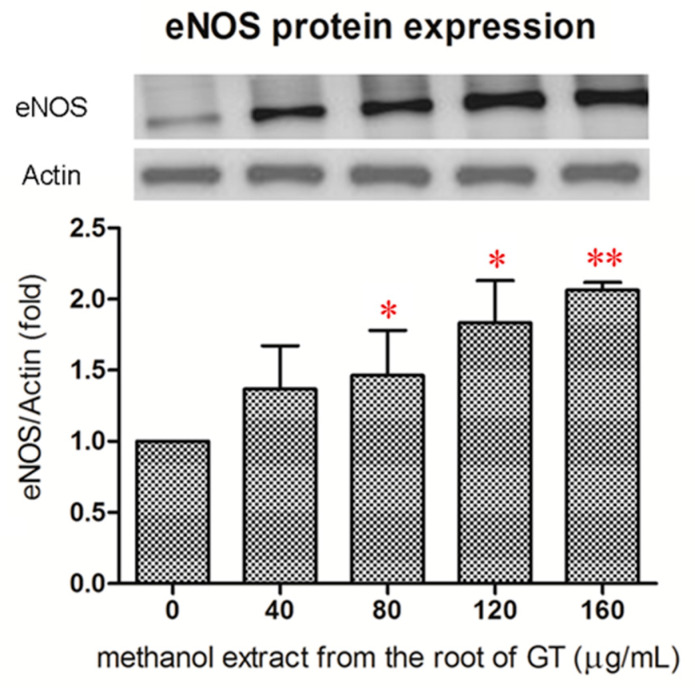
Effect of the R/M GTE on eNOS protein expression in HUVECs. HUVECs was treated with R/M extract for 24 h. Total eNOS protein content was determined by Western blot analysis, using β-actin as an internal control. Data were obtained from four replicate experiments, and are expressed as mean ± standard deviation. A significant difference from the vehicle was indicated as * *p* < 0.05 or ** *p* < 0.01 (Student’s *t*-test).

Since the R/M GTE exhibited the most potent effect on NO production, we then examined if the R/M GTE increased the expression of eNOS. As analyzed with Western blot, treatment of HUVECs with R/M GTE for 24 h resulted in a significant increase in eNOS protein expression at a dose higher than 80 μg/mL (Figure 3).

### 2.4. Effect of the R/M GTE on eNOS mRNA Expression

**Figure 4:**
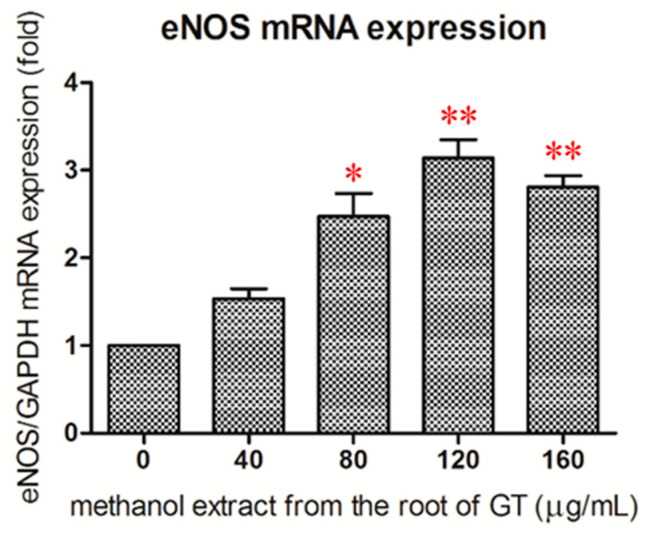
Effect of R/M GTE on eNOS mRNA expression in HUVECs. HUVECs were treated with R/M extract for 24 h. Real-time RT–PCR analysis was performed in order to analyze the expression level of mRNA of eNOS. Data were obtained from triplicate experiments, and are expressed as mean ± standard deviation. A significant difference from the vehicle was indicated as * *p* < 0.05 or ** *p* < 0.01 (Student’s *t*-test).

Real-time RT–PCR analysis revealed that treatment of HUVECs with the R/M GTE for 24 h resulted in a significant increase in eNOS mRNA expression at a dose higher than 80 μg/mL (Figure 4). This indicated that the R/M GTE increased the expression of eNOS at the gene transcription level of HUVECs.

### 2.5. Effect of the R/M GTE on eNOS Phosphorylation at Ser^1177^

**Figure 5:**
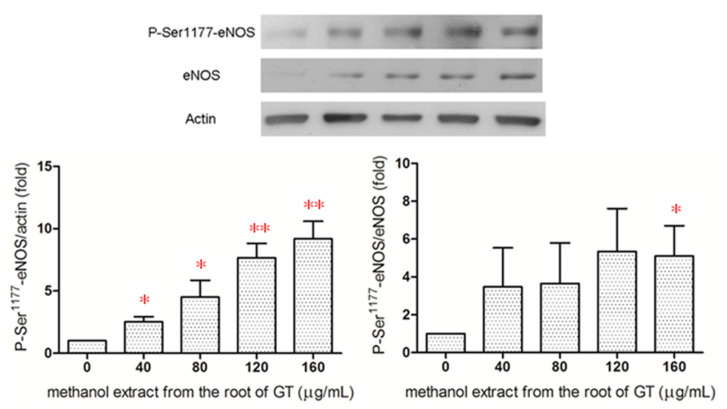
Effect of the R/M GTE on eNOS activation. HUVECs were treated with R/M extract for 15 min. Total eNOS protein content and phospho-eNOS (P-Ser^1177^-eNOS) were determined by Western blot analysis. Data were obtained from four replicate experiments, and are expressed as mean ± standard deviation. A significant difference from the vehicle was indicated as * *p* < 0.05 or ** *p* < 0.01 (Student’s *t*-test).

Phosphorylation of eNOS at Ser^1177^ activates eNOS [28]. Western blot analysis revealed that the R/M GTE increased eNOS phosphorylation at serine^1177^ dose-dependently (Figure 5), and in as early as 15 min after treatment of HUVECs (Supplementary Figure S2). The ratio of P-Ser^1177^-eNOS/eNOS also increased after treatment of HUVECs with the R/M GTE for 15 min. The results indicated that the R/M GTE increased the activity of eNOS in HUVECs.

### 2.6. Chemical Composition Analysis

**Figure 6:**
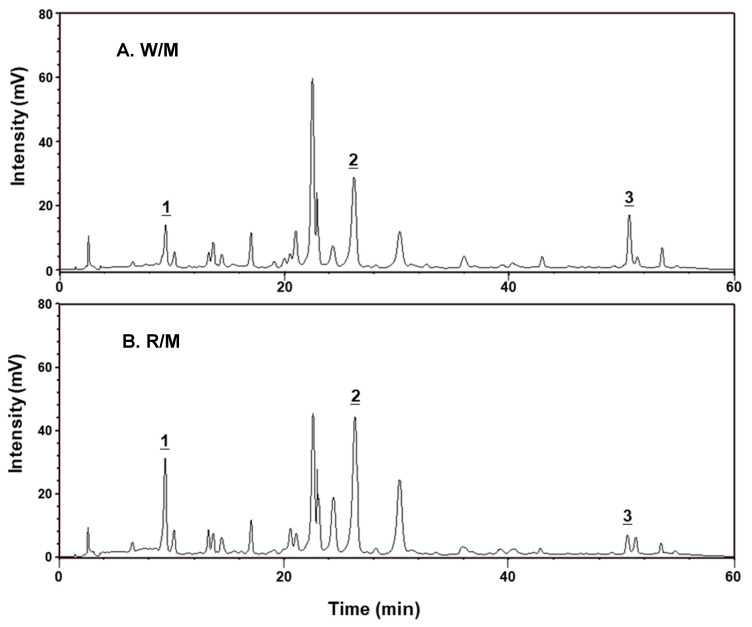
HPLC chromatogram of the W/M GTE (**A**) and R/M GTE (**B**). The mobile phase was methanol/0.05% acetic acid (4:6, *v*/*v*) with the flow rate of 1.0 mL/min. The detection was performed at 350 nm. Identified components: 1, chlorogenic acid; 2, luteolin-7-glucoside; and 3, luteolin.

The main active ingredients of the GT extracts are polyphenol and flavonoid compounds [29]. Figure 6 shows the HPLC chromatograms of the R/M GTE and the methanol extract of the whole GT plant (W/M GTE). Three components were identified as chlorogenic acid (CGA, peak 1, retention time (RT) = 9.6 min), luteolin-7-glucoside (lut-7-g, peak 2, RT = 26.2 min), and luteolin (lut, peak 3, RT = 50.6 min). Table 1 shows that the R/M GTE had higher amounts of CGA and lut than those of the W/M GTE. In addition, the contents of total polyphenols and total flavonoids of the R/M GTE were also higher than those of the W/M GTE.

## 3. Discussion

The present study demonstrated that methanol and ethanol extracts of GT increased endothelial NO synthesis. The R/M GTE exhibited the most potent effect on NO production. Incubation with the R/M GTE increased the protein and mRNA expression of eNOS in HUVECs. Furthermore, the R/M GTE activated eNOS by the phosphorylation of eNOS at Ser^1177^.

In the blood vessels, eNOS-derived NO possesses vasodilatative, anti-thrombotic, and anti-atherosclerotic effects [2,5,30]. Impairment of endothelial NO bioactivity resulted in endothelial dysfunction, i.e., the earliest event of atherosclerosis [4]. Some studies have shown that deficiency of eNOS enhances atherosclerosis [5,31]. However, animal studies have demonstrated the unchanged or rather augmented expression of eNOS in atherosclerosis [32,33]. This suggested that the upregulation of eNOS expression does not necessarily increase the production of NO. Under pathological conditions, oxidative stress increases superoxide production in the atherosclerotic lesion. Superoxide reacts with NO to form peroxynitrite and reduce its bioactivity. Peroxynitrite further oxidizes tetrahydrobiopterin (BH_4_) to 7,8-dihydrobiopteritin (BH_2_). The deficiency of BH_4_ results in the phenomenon known as “eNOS uncoupling”. Under this condition, eNOS is dysfunctional, and the enzymatic reduction of molecular oxygen by eNOS is no longer coupled to L-arginine oxidation, resulting in the production of superoxide rather than NO [2,34].

In the present study, the methanol extract of GT exhibited more potent effects on NO production than other solvent extracts. It seems that methanol can extract more bioactive substances that can promote NO production. As reported, the antioxidant activity of many edible plant extracts, fruit, and vegetables increases with their polyphenol content [35,36,37]. Tsai et al. demonstrated that the total polyphenols and flavonoids content were highest in methanol extract, which is a high polarity extract from GT [29]. Additionally, they found that the antioxidant activities of GT declined in the order of methanol extract > EA extract > hexane extract. However, there is a discrepancy between the findings of Tsai et al. and the present study. Whereas the former found that the aerial part of GT had superior antioxidant activity to the other part of extracts, the latter has demonstrated that the R/M GTE had a more potent effect on NO production than that from other parts of GT. Therefore, it is assumed that the antioxidant property of the methanol extract of GT does not play a primary role in the mechanism of increasing endothelial NO production.

Three major bioactive ingredients of GT have been reported [27], of which CGA is an ester of caffeic acid and (−)-quinic acid, and lut and lut-7-g are flavonoids. CGA was demonstrated to have protective effect on endothelial vessel function against oxidant-induced damage via the increased production of NO and induction of Heme oxygenase-1 (Hmox-1) [38]. CGA administration attenuated vascular senescence in a dose-dependent manner through the Nrf2/HO-1 pathway and the increase of eNOS expression in an in vivo model [39]. In Type I diabetic mice, CGA treatment increased cyclic GMP (cGMP) level and activated protein kinase G (PKG) in cardiac fibroblasts by enhancing eNOS activity and NO production, thereby contributing to the inhibition on cardiac fibrosis caused by hyperglycemia [40].

Lut upregulated eNOS expression by increasing eNOS promoter activity and eNOS mRNA expression in human endothelial cells [41]. In a diabetic rat model, lut was revealed to have a protective effect on the diabetic heart against myocardial ischemia/reperfusion injury by upregulating the myocardial eNOS pathway, Nrf2 and the Nrf2-related antioxidative signaling pathway, the enhancement of manganese superoxide dismutase (MnSOD) activity, and inhibition of the mitochondrial permeability transition pore (mPTP) [42,43]. Lut exhibited vasorelaxation in endothelial intact vessels and phenylephrine or potassium-contracted aortic rings, inhibited CaCl_2_-induced contraction in endothelial denuded arterial rings, and attenuated transient contraction by the stimulation of NO-dependent vascular dilatation [44,45]. In addition, lut was shown to have the beneficial effects on cardiometabolic alterations and vascular dysfunction in mice with high fat diet-induced obesity [46] as well as on mitochondrial dysfunction in endothelial cells [47]. Furthermore, our previous paper reported that lut and lut-7-g were effective in preventing the adhesion of monocytes to cytokine-activated endothelium by the inhibition of expression of adhesion molecules, and that they had therapeutic potential for atherosclerosis [26].

Comparing the chromatograms of R/M and W/M extracts from the HPLC analysis shown in Figure 6, there is little difference in the components contained in the two extracts. Although the R/M GTE contained a slightly higher active ingredient content than the W/M GTE, the NO production of the R/M GTE was significantly higher than that of the W/M GTE (Figure 2). The NO production of the R/Et GTE was also higher than other extracts. Since the present HPLC analysis method can only analyze a part of the polar substances, especially flavonoid compounds, other unidentified bioactive components of the root, which may be responsible for the eNOS upregulation and activation, need to be further explored. 

The present study has demonstrated that the R/M GTE upregulated the expression of eNOS. Additionally, it activated eNOS by phosphorylation of eNOS at ser^1177^ as early as 15 min after treatment. Although many studies have reported that all three of the main bioactive ingredients of GT, CGA, lut, and lut-7-g, possess beneficial effects on endothelial function, the root extract of GT may contain other active ingredients that require further investigation. In combination with good antioxidant activity of the high polarity methanol extracts of GT, the R/M GTE enhanced NO production, and provided a potential therapeutic remedy for vascular protection and prevention of atherosclerotic cardiovascular disease.

## 4. Materials and Methods

### 4.1. Materials

The raw materials of GT were bought from an herb store in Penghu Island, Taiwan. The preparation of each extract of GT was described previously [29]. In brief, the dried whole plant, aerial, and root of GT (200 g each) were crushed and drenched in 500 mL EA, *n*-hexane, ethanol, and methanol, respectively, for one day and then extracted three times with 500 mL of the same solvent by stirring at room temperature. After filtration by medicinal gauze, the filtrates were concentrated with a vacuum evaporator and dried with a freeze-drier. Each extract was dissolved in dimethyl sulfoxide (DMSO) to make fresh stock solutions before each assay. The final culture concentration of DMSO was 0.25%. Penicillin, streptomycin, DMSO, and 3-(4,5-dimethylthiazol-2-yl)-2,5-diphenyltetrazolium bromide (MTT) were purchased from Sigma-Aldrich Chemicals (St Louis, MO, USA). All other chemicals used were of reagent or analytical grade.

### 4.2. Cell Cultures

HUVECs were obtained from Lonza (Walkerville, MD, USA) and maintained in endothelial cell basal medium (EBM-2) supplemented with endothelial cell growth medium EGM-2 (Lonza, Basel, Switzerland) at 37 °C under 5% CO_2_ atmosphere. HUVECs with passage between 4 and 6 were used in the study.

### 4.3. MTT Assay for Cell Viability

HUVECs were seeded into 96 well plates at 2 × 10^4^ cells per well and treated with the indicated concentration of each extract of GT. The cells were cultivated at 37 °C with 5% CO_2_ and 95% air, at 100% relative humidity. After 24 h of incubation, the medium solution was removed. An aliquot of 10 μL of 5 mg/mL MTT was added to the plates and the cells were incubated for 4 h. After removing the medium solution, 100 µL of DMSO were added to each well to dissolve the formazan crystals formed, which was shaken until the crystals dissolved. The cytotoxicity against HUVECs was determined by measuring the absorbance of the converted dye at a wavelength of 570 nm in a fluorescence microplate reader (Sunrise, Tecan, Mannedorf, Switzerland). Cell viability (% of control) was calculated as (experimental OD/control OD) × 100%.

### 4.4. Determination of NO Synthesis

NO production was determined as the formation of nitrite. HUVECs were seeded at a density of 1 × 10^5^ cells/well into a 24 well plate coated with gelatin (Sigma-Aldrich) for NO production. The cells were starved in serum-free medium for 1 h and treated with each extract at indicated concentration for 24 h. At the end of incubations, the supernatants of the culture were sampled, and left over night at 4 °C for conversion of NO to nitrate and nitrite. Nitrate was completely oxidized to nitrite by adding nitrate reductase into the supernatants for 2 h. Following the Griess reaction, the amount of nitrite was determined by measuring the absorbance at a wavelength of 540 nm in a microplate reader (Tecan Sunrise, Switzerland).

### 4.5. Western Blot Analysis

Treated HUVECs were scraped off from the dish and lysed with RIPA buffer (Roche, Basel, Switzerland), which consists of 50 mM Tris–HCl (pH 7.4), 150 mM NaCl, 0.5% sodium deoxycholate, 1% NP-40, 1% SDS, and protease inhibitor mixture for eNOS analysis or combined protease and phosphatase inhibitor mixtures for phospho-eNOS analysis, at 4 °C overnight. The protein concentration of the cell lysate was quantified using the Bradford Protein Assay Kit (Bio-Rad, Hercules, CA, USA). Equal amounts of protein from each sample were applied to SDS-polyacrylamide gel electrophoresis (SDS–PAGE) and then transferred to a PVDF membrane (Bio-Rad). The membrane was blocked with 5% skim milk and incubated with the purified mouse anti-eNOS Type III or anti-eNOS (pS1177) (BD Biosciences, Franklin Lakes, NJ, USA) at 4 °C overnight. The blots were then probed with the secondary antibody linked to peroxidase (Jackson ImmunoResearch, West Grove, PA, USA). Immunoreactive proteins were detected using a chemiluminescent kit (Luminata Crescendo Western HRP Substrate) (Millipore, Billerica, MA, USA). The intensity of each band was measured by densitometry (UVP BioSpectrum 500 Imaging System, Upland, CA, USA).

### 4.6. Real-Time RT-PCR Analysis 

Total cellular mRNA was extracted from the treated HUVECs using Trizol reagent (Invitrogen). An amount of 2 μg RNA of each sample was reversibly transcripted into cDNA. The quantitative real-time PCR assay was carried out in a 48 well plate using the Eco Real-Time PCR System (Illumina, San Diego, CA, USA) and the optimized standard SYBR Green 2-step qRT–PCR kit protocol (Thermo Scientific Maxima SYBR Green qPCR Master Mix, Waltham, MA, USA). PCR primers used were as follows: eNOS, sense: 5′-TCTCCGCCTCGCTCATG-3′, anti-sense: 5′-AGCCATACAGGATTGTCGCC-3′; GAPDH, sense: 5′-CAACGGATTTGGTCGTATT-3′, anti-sense: 5′-ATATTGGAACATGTAAACCATGTA-3′. GAPDH was used as an endogenous control to normalize the input amount of each sample.

### 4.7. Chemical Composition Analysis

The main ingredients composition analysis by HPLC and the determination of the total polyphenols content and total flavonoids content were conducted according to the methods of our previous paper [27]. In brief, the amounts of main components of GT extracts were determined by Ascentis^TM^ C18 column (No. 581325-U, 5 μm, 250 × 4.6 mm; Supelco, Bellefonte, PA, USA) in an HPLC (Model L-7100, Hitachi, Tokyo, Japan) with a UV/Visable detector (Model L-7420, Hitachi). The mobile phase was methanol/0.05% acetic acid in water (4:6, *v*/*v*) with the flow rate of 1.0 mL/min. The detection was performed at 350 nm. The compounds used to build the calibration curves were purchased from Sigma-Aldrich.

For the determination of total polyphenols content, an extract sample of 0.15 mL was mixed with 0.75 mL of 0.2 N Folin–Ciocalteu reagent (Sigma-Aldrich) and 0.6 mL of 7.5% (*w*/*v*) Na_2_CO_3_ solution. The mixture was kept at room temperature for 30 min and then the absorbance was spectrophotometrically measured at 765 nm. The total polyphenols content was expressed as gallic acid equivalents of dry extract.

To determine the total flavonoids content, an extract sample of 0.3 mL of the sample was mixed with 1.2 mL of water and 0.075 mL of 15% (*w*/*v*) Na_2_CO_3_. After 5 min, 0.15 mL of 10% (*w*/*v*) AlCl_3_ was added and kept at room temperature for 6 min. After that, 0.5 mL of 1 M NaOH and 0.275 mL of deionized water were added and vigorously shaken. The absorbance was spectrophotometrically measured at 510 nm. The total flavonoids content was expressed as catechin equivalents of dry extract.

### 4.8. Statistical Analysis

Data of each result were obtained from three to five independent experiments, each experiment in triplicate. Statistical differences were analyzed by Student’s *t*-test, one-way ANOVA analysis, and post-hoc analysis (* *p* < 0.05, ** *p* < 0.01 or *** *p* < 0.001). The experimental data were analyzed using SPSS Statistics software (Version 18.0, IBM Co., Armonk, NY, USA).

## 5. Conclusions

The present study demonstrated that methanol and ethanol extracts of GT increased endothelial NO synthesis. The R/M GTE upregulated the expression of eNOS, activating eNOS by phosphorylation of eNOS at ser^1177^, and exhibiting the most potent effect on NO production. It provided a potential therapeutic remedy for vascular protection and the prevention of atherosclerotic cardiovascular disease. 

## Figures and Tables

**Table 1:** Main ingredients analysis of the W/M GTE and R/M GTE *.

Ingredient	Concentration (mg/g Extract)	
	W/M GTE	R/M GTE
Chlorogenic Acid (1)	5.32 ± 0.13	8.59 ± 0.31
Luteolin-7-glucoside (2)	44.51 ± 0.27	63.02 ± 0.69
Luteolin (3)	4.23 ± 0.28	2.10 ± 0.11
Total Polyphenols Content	61.78 ± 0.84	76.72 ± 1.27
Total Flavonoids Content	58.56 ± 1.50	66.44 ± 1.68

* Data were obtained from triplicate experiments, and are expressed as mean ± standard deviation.

## Data Availability

Data is contained within the article and supplementary material.

## Supplementary Materials

The following are available online at https://www.mdpi.com/article/10.3390/ph14060577/s1, Figure S1: Effects of different extracts from the whole plant, aerial and root of GT on the proliferation of HUVECs. The treatment time was 24 h. The cell viability was measured by an MTT assay kit, and the number of cells was regarded as 100% for the vehicle group. Data were estimated from five replicate experiments. W: whole plant; A: aerial; R: root; E: EA extract; H: hexane extract; Et: ethanol extract; M: methanol extract, Figure S2: Effect of the treatment time of R/M GTE on eNOS activation. HUVECs was treated with R/M extract for 0-24 h. The phospho-eNOS (P-Ser1177-eNOS) contents were determined by Western blot analysis.
