# Supplementary material for: Glossogyne tenuifolia Extract Increases Nitric Oxide Production in Human Umbilical Vein Endothelial Cells
Source: Pharmaceuticals (Basel). 2021 Jun 17;14(6):577. doi: 10.3390/ph14060577 (PMC8235410; doi:10.3390/ph14060577)
Supplement: Supplementary file 1 [file pharmaceuticals-14-00577-s001.zip › pharmaceuticals-1243914-supplementary.pptx]

## Slide 1
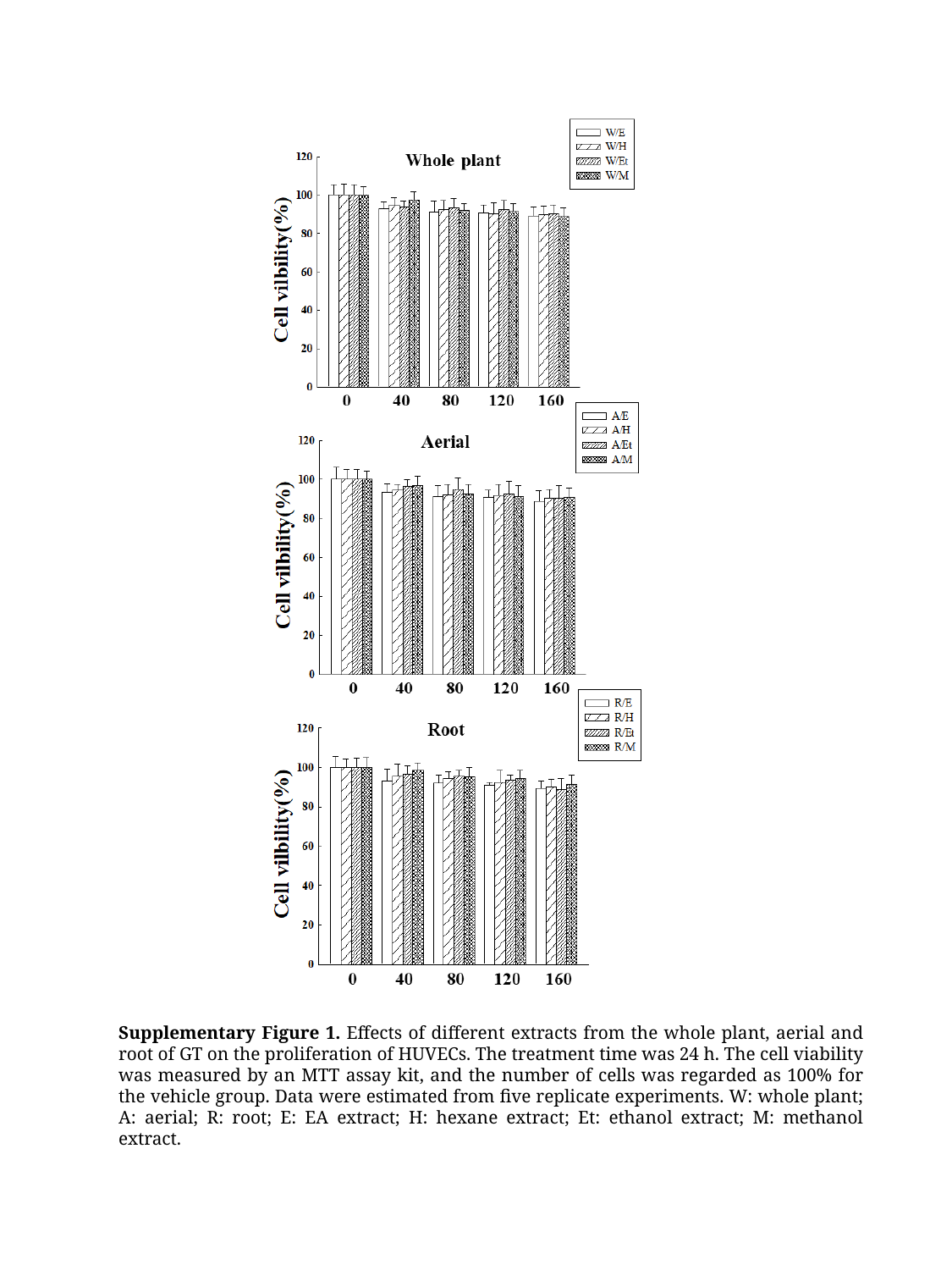

Supplementary Figure 1. Effects of different extracts from the whole plant, aerial and root of GT on the proliferation of HUVECs. The treatment time was 24 h. The cell viability was measured by an MTT assay kit, and the number of cells was regarded as 100% for the vehicle group. Data were estimated from five replicate experiments. W: whole plant; A: aerial; R: root; E: EA extract; H: hexane extract; Et: ethanol extract; M: methanol extract.

## Slide 2
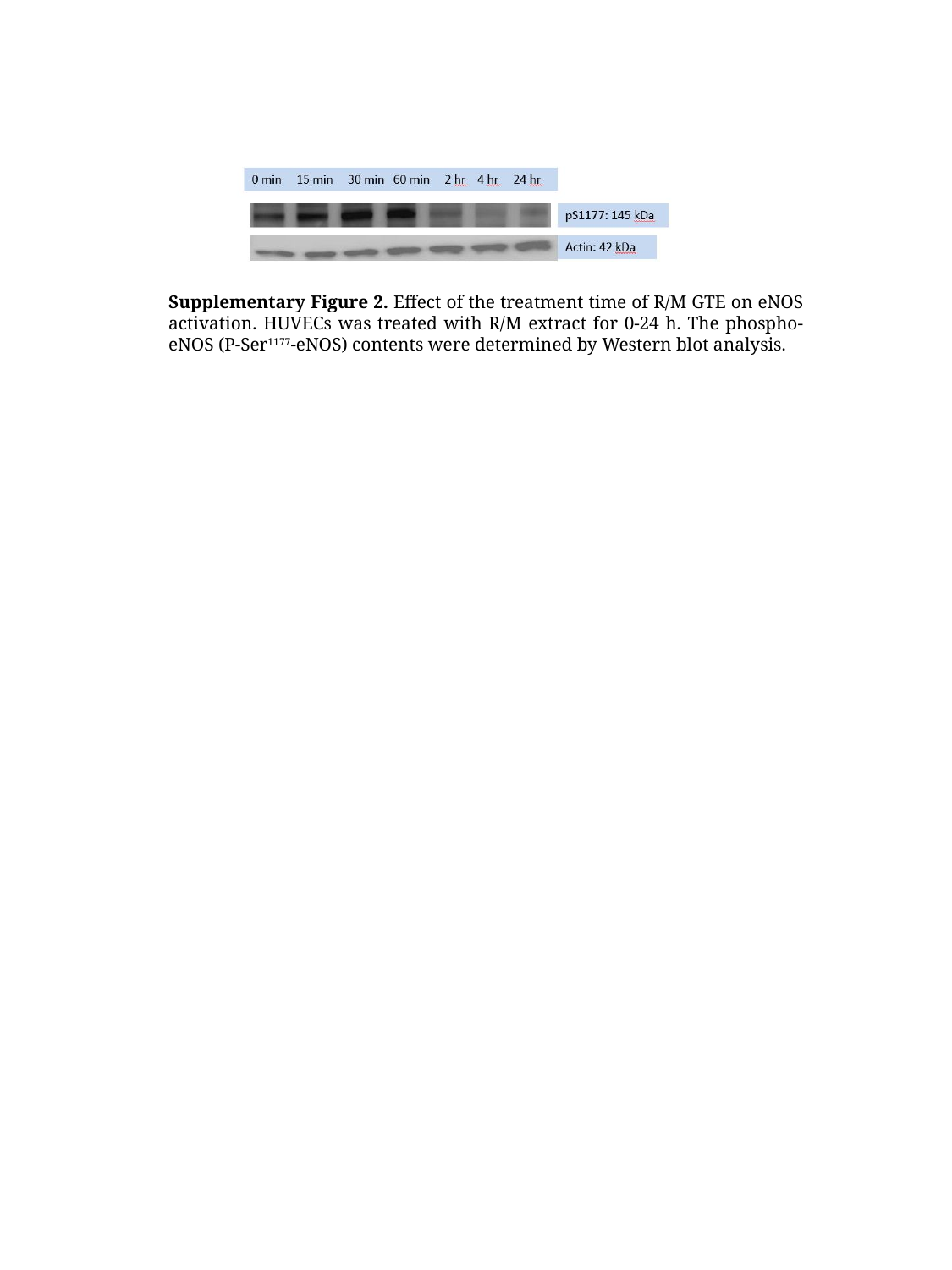

Supplementary Figure 2. Effect of the treatment time of R/M GTE on eNOS activation. HUVECs was treated with R/M extract for 0-24 h. The phospho-eNOS (P-Ser1177-eNOS) contents were determined by Western blot analysis.
